# Dental Emergency Admissions in Emergency Oral Health Care Centers during COVID-19 Pandemic in Buenos Aires, Argentina

**DOI:** 10.3390/ijerph19031528

**Published:** 2022-01-29

**Authors:** Pablo Alejandro Rodriguez, Patricio Gatti, María Lorena Cabirta, Nicolas Roman Baquerizo, Silvio Prada, Ariel Gualtieri, Sebastian Puia, Aldo Squassi

**Affiliations:** 1 Universidad de Buenos Aires, Facultad de Odontología, Cátedra de Endodoncia, Buenos Aires 1122, Argentina; prodriguez@odontologia.uba.ar (P.A.R.); lorena.cabirta@odontologia.uba.ar (M.L.C.); 2 Universidad de Buenos Aires, Facultad de Odontología, Cátedra de Cirugía y Traumatología BucoMaxiloFacial I, Buenos Aires 1122, Argentina; patricio.gatti@odontologia.uba.ar (P.G.); sebastian.puia@odontologia.uba.ar (S.P.); 3 Universidad de Buenos Aires, Facultad de Odontología, Servicio de Urgencias y Orientación de Pacientes, Buenos Aires 1122, Argentina; silvio.prada@yahoo.com; 4 Universidad de Buenos Aires, Facultad de Odontología, Cátedra de Biofísica y Bioestadística, Buenos Aires 1122, Argentina; gualtieriariel@gmail.com; 5 Universidad de Buenos Aires, Facultad de Odontología, Cátedra de Odontología Preventiva y Comunitaria, Buenos Aires 1122, Argentina; 6 Universidad de Buenos Aires, Facultad de Odontología, Instituto de Investigaciones en Salud Pública, Buenos Aires 1122, Argentina; 7 Universidad de Buenos Aires, Facultad de Odontología, Cátedra de Bioquímica, Buenos Aires 1122, Argentina; 8 Dental Servicios Odontológicos, Caseros, Buenos Aires 1678, Argentina; endodoncia@odontologia.uba.ar; 9 Universidad de Buenos Aires, Facultad de Odontología, Cátedra de Cirugía y Traumatología BucoMaxiloFacial II, Buenos Aires 1122, Argentina

**Keywords:** COVID-19, dental health services, delivery of health care, dental emergency, Argentina, global pandemic, Sars-CoV-2

## Abstract

This study aimed to describe patients’ demographic characteristics and treatment delivered in private and public dental emergency clinics and to compare them with a pre-pandemic period. A retrospective cross-sectional study was carried out on patients attending the University Dental Hospital of the University of Buenos Aires and on one private dental clinic, situated in Buenos Aires, between March and June of 2019 and 2020. The sample considered all the patients’ medical records of those requiring emergency dental treatment at these centers during that period. We systematize the treatments required and delivered by the emergency providers into categories. In 2019, 36,071 patients were treated at the university dental hospital and the private clinic. In 2020, 7485 patients received emergency treatment, showing a fivefold reduction. During a pre-pandemic period, in 2019, both oral care centers provided 44,894 treatments, around three times the treatments provided in 2020 during the same period. We have found significant differences in the age groups of people who required emergency treatment in the pandemic period compared to the pre-pandemic period. During the pandemic period, the most represented types of treatment were radiographic exams (3061) and tooth extractions (2583). Conversely, during the pre-pandemic period, the most frequent treatments were radiographic examinations (16,649) and general consultations (10,591). The percentages of all types of treatments differ significantly between the two years except for pediatric consultations and prosthetics emergencies. As the pandemic is still an ongoing sanitary problem, urgent dental care patterns should be considered.

## 1. Introduction

The coronavirus disease 2019 (COVID-19) pandemic was first reported in Hubei Province, China, in December 2019 [1]. On January 22, an epidemiological alert was issued in Argentina, and surveillance of the event began. On March 3, through the National Surveillance System in Health (SNVS 2.0), the first confirmed case was reported in the Buenos Aires Metropolitan Area [2].

In different countries, governmental authorities implemented multiple public health tools and interventions to minimize morbidity and mortality related to COVID-19, including extreme public health measures, such as lockdowns and stay-at-home mandates, that were initially critical to flattening the curve [3].

Argentina has adopted varied social-distancing preventive interventions and related socio-economic decisions since 10 March 2020 [4]. First, a presidential decree (N° 297/2020) established that Preventive Mandatory Social Isolation (PMSI) became mandatory for all Argentinean inhabitants from 20 to 31 March 2020. However, on 29 March, the government announced the first quarantine extension until 13th April. Then, on 10 April, the government implemented a second extension until 26 April, and subsequently, several additional extensions were implemented, reaching a quarantine duration of 285 days. However, at the end of July, the number of cases increased dramatically, being circa 6000 new cases per day.

Since the risk of infection during diagnosis and treatment of oral diseases was considered high [5], sanitary authorities suspended scheduled dental care during PMSI at most private and social security oral healthcare centers in the Buenos Aires Metropolitan Area, which concentrates 37% of the population in Argentina (approximately 14,800,000 inhabitants). 

In developed countries, university dental hospitals and private clinics postponed and canceled scheduled visits at general dentistry and specialty clinics and adopted policies to provide only urgent care following the national guidelines. The Eastman Institute of Oral Health reported a reduction of 85% of consultations since the beginning of restrictions in the United States [6]. Izzeti et al. reported that dental activity was reduced in Italy by 95% and limited to urgent care such as treatment of pulpitis, dental abscess, and prosthesis re-cementation [7]. Cagetti et al. studied the relationship between the number of admissions for urgent dental care and the different phases of the COVID-19 pandemic. They reported a significant reduction in consultations during the lockdown, increasing during the reopening, and that tooth extraction was the most frequently delivered treatment [8]. Demand patterns during the pandemic may have been affected by geographic barriers and by the perception of patients regarding differences in care between a public hospital and a private practice. Previous studies have not analyzed these variables, and we included them in the present work. Since the age of the patients could be an intervening variable in the decision to carry out an emergency dental visit, it is also essential to incorporate this demographic variable. 

The University Dental Hospital of the School of Dentistry of the University of Buenos Aires remained open permanently, providing dental care from the beginning of the pandemic to date, mainly to patients from low-income neighborhoods. Furthermore, its Emergency Department has a seven-operatory clinic dedicated to treating patients with dental emergencies and requiring urgent care. Besides, in the metropolitan area (3800 km^2^), some private dental clinics remained open, providing emergency care to the surrounding population. 

This study aimed to describe patients’ demographic characteristics and treatment delivered in private and public dental emergency clinics and compare them with a pre-pandemic period.

## 2. Materials and Methods

A retrospective cross-sectional study was carried out on patients attending the Emergency and Patient Orientation Service of the University Dental Hospital of the School of Dentistry of the University of Buenos Aires (EPOS), located in Buenos Aires city, and on one private dental clinic, “Dental Servicios Odontológicos” (DSO), situated in the Metropolitan Area of Buenos Aires, in the period between March 20 and June 20 of the years 2019 and 2020.

The study was designed to compare the oral health services provided by the EPOS and DSO in two different periods of three months each: March 20th to June 20th of 2019 (pre-pandemic period) and March 20th to June 20th of 2020 (pandemic period).

The sample considered all the medical records prepared for the patients who attended during that period for dental emergency issues. All admitted patients received dental treatment to resolve the emergencies that had generated the demand for care.

In both centers, the care protocol included a process that comprised three stages:

1. Emergency diagnostic process: includes preparing a general medical history and performance of the oral exam.
2. Emergency therapeutic process: includes the implementation of standardized practices according to the diagnosis.
3. Patient orientation: includes referral of the patient to different public or private services following a comprehensive treatment plan designed after the resolution of the dental emergency.

The entire process was carried out by dentists trained in emergency diagnosis and treatment, who recorded data in a single clinical history program designed for this purpose in both institutions, as well as age and sex of patients 

Different delivered treatments were categorized into groups: general dental consultation; pediatric consultation; tooth extraction (simple); oral surgery; emergency cementation of dental restorations; follow-ups; endodontic treatments; oral medicine consultation; conservative treatment (restorations); radiography (periapical, bitewing, panoramic); traumatisms; pericoronitis; periodontal treatments; and prosthetic treatment. 

Studies based on hospital registers do not require further Ethics Committee approval to comply with Argentine regulations. This study did not receive any funding

The description of age included the following measures: mean, standard deviation (SD), median, first quartile (Q1), third quartile (Q3), minimum and maximum.

We described categorical data using absolute frequencies (AF) and percentages. 95% confidence intervals (95% CI) were estimated for percentages using the score method [9]. Pearson’s Chi-square test was used to compare frequencies. In addition, to analyze the association between the provision and the place of care, Cramer’s V coefficient was evaluated [10]. We also made post-hoc comparisons based on the residual analysis of Pearson’s Chi-square test [11], with Bonferroni correction. A significance level of 5% was set. The following statistical software were used: Calc, from Apache OpenOfficeTM v. 4.1.6 [12], Infostat v. 2020 [13], and R v. 4.0.3 [14] with rcompanion [15] and chisq.posthoc.test [16] packages. 

## 3. Results

In the three months of 2019, 36,071 patients (11,835 female (32.8%; CI95: 32.3–33.3%) and 24,236 male (67.2%; CI95: 66.7–67.7%)) were treated at the university dental hospital and the private clinic. Meanwhile, in 2020, 7485 patients (2730 female (36.5%; CI95: 35.4–37.6%) and 4755 male (63.5%; IC95: 62.4–64.6%)) received emergency treatment, showing a fivefold reduction. In both cases, differences between sex were significant (χ^2^ = 4263.39; *p* < 0.05 in 2019; and χ^2^ = 547.85; *p* < 0.05 in 2020). A significant association was found between sex and year of consultation (2019 vs. 2020) (Chi-square = 37.36; *p* < 0.05). However, despite being significant, it was a negligible association (Cramer’s V = 0.03).

In 2019, the age of the patients was between 3 and 108 years old, with a median (Q1; Q3) of 42 years (29; 57) and a mean (SD) of 44 years (19). In 2020, the age of the patients ranged from 1 to 99 years, with a median (Q1; Q3) of 37 years (28; 50) and a mean (SD) of 39 years (16). The distribution of patients according to age group is represented in Table 1. 

In 2020, this distribution differs significantly from a uniform distribution (Chi-square = 4280.97; *p* < 0.05). The most represented groups were 30 to 39 years, with 1855 patients (24.8%; 95 CI: 23.8–25.8%) and 20 to 29 years, with 1594 patients (21.3%; 95 CI: 20.4–22.2%). The distribution within each sex was similar to the global distribution. The age of the women was between 1 and 92 years old, with a median (Q1; Q3) of 37 years (27; 49) and a mean (SD) of 38 years (17). Among men, the age was between 1 and 99 years, with a median (Q1; Q3) of 38 years (28, 51) and a mean (SD) of 40 years (16). In 2019, this distribution also differs significantly from a uniform distribution (Chi-square = 11,733.47; gl = 8; *p* < 0.05). As in 2020, the most highly represented groups were 30 to 39 years (7172 patients; 19.9%; 95 CI: 19.5–20.3%) and 20 to 29 years (6837 patients; 19.0%; 95 CI: 18.6–19.4%).

A significant association was found between age group and year (pre-pandemic vs. pandemic year) (Chi-square = 548.33; *p* < 0.05), although Cramer’s V coefficient was 0.11, which indicates a weak association. Post hoc comparisons indicate that the percentages of all age groups differ significantly between the two years (*p* < 0.05), except for the group aged 50 to 59 years (2019/2020; 14.7%/13.3%). The age groups (years old) 10–19, 60–69, 70–79 and ≥80 had higher percentages in 2019, *p* < 0.05 (age group: 2019/2020; 10–19: 5.8%/4.7%; 60–69: 10.9%/8.1%; 70–79: 7.5%/3.5%; ≥80: 3.5%/0.9%). In contrast, the age groups (years old) ≤ 9, 20–29, 30–39 and 40–49 had higher percentages in 2020, *p* < 0.05 (age group: 2019/2020: ≤9: 2.1%/3.8%; 20–29: 19.0%/21.3%; 30–39: 19.9%/24.8%; 40–49: 16.6%/19.7%).

A significant association was found within the private clinic between age group and year of consultation (Chi-square = 728.04; gl = 8; *p* < 0.05), with a Cramer’s V coefficient of 0.22, which indicates a moderate association. Post hoc comparisons indicate that, within the private clinic, the percentages of all age groups differ significantly between both years (*p* < 0.05), except for the group aged 50 to 59 years (2019/2020; 13.1%/11.2%). The age groups (years) 10–19, 60–69, 70–79 and ≥80 had higher percentages in 2019, *p* < 0.05 (age group: 2019/2020; 10–19: 10.8%/8.3%; 60–69: 12.8%/7.7%; 70–79: 11.2%/2.7%; ≥80: 7.4%/0.6%). In contrast, the age groups (years) ≤9, 20–29, 30–39 and 40–49 had higher percentages in 2020, *p* < 0.05 (age group: 2019/2020: ≤9: 5.6%/8.8%; 20–29: 12.2%/16.9%; 30–39: 13.7%/24.2%; 40–49: 13.1%/19.6%).

Within the university hospital, a significant association was also found between age group and year (Chi-square = 69.61; gl = 8; *p* < 0.05, although Cramer’s V coefficient was 0.05, which indicates a negligible association. Post hoc comparisons indicate that, within the hospital, only the percentages of the following three age groups (years) differ significantly between the two years (*p* < 0.05): ≤9, 60–69 and 70–79. The ≤9 group had a higher percentage in 2020, while the 60–69 and 70–79 groups had higher percentages in 2019, *p* < 0.05 (age group: 2019/2020; ≤9: 0.3%/0.7%; 60–69: 9.9%/8.3%; 70–79: 5.6%/4.0%). For all the other age groups there were no significant differences (age group: 2019/2020; 10–19: 3.1%/2.5%; 20–29: 22.5%/24.0%; 30–39: 23.1%/25.1%; 40–49: 18.5%/19.7%; 50–59: 15.5%/14.6%; ≥80: 1.4%/1.1%).

The number of treatments performed on patients in the pandemic period was 14,161. The distribution according to type of treatment differs significantly from a uniform distribution (Chi-square = 14,097.43; *p* < 0.05), and the most represented types were radiographs and tooth extraction (3061 radiographic exams (21.6%; 95 CI: 20.9–22.3%) and 2583 tooth extractions (18.2%; 95 CI: 17.6–18.9%)). The distribution of treatments according to place of delivered care during pandemic period is represented in Table 2. 

During a pre-pandemic period, in 2019, both oral care centers provided 44,894 treatments, around three times the treatments provided in 2020 during the same period. In that year, taking the private clinic and university hospital data together, the distribution according to type of treatment also differs significantly from a uniform distribution (Chi-square = 102,064.11; *p* < 0.05. The most highly represented were radiograph examinations and general consultations (16,649 radiographic examinations (37.1%; 95 CI: 36.6–37.5%) and 10,591 general consultations (23.6%; 95 CI: 23.2–24.0%)). The distribution of treatments according to place of delivered care during the pre-pandemic period is represented in Table 3. 

A significant association was found between the treatment and the year (Chi-square = 8407.20; *p* <0.05. Cramer’s V coefficient was 0.38, indicating a moderate association. Post hoc comparisons indicate that the percentages of all types of treatments differ significantly between the two years except for pediatric dentistry (2019/2020; 1.0%/0.8%) and prosthetics (2019/2020; 3.4%/3.1%). The percentages of general consultations, radiographic exams, periodontics and restorations were higher in 2019, *p* < 0.05 (treatment: 2019/2020; general consultations: 23.6%/11.4%; radiographic exams: 37.1%/21, 6%; periodontics: 5.4%/1.0%; restorations: 11.0%/5.4%). The percentages of cementation, oral surgery, follow-ups, endodontics, oral medicine consultation, tooth extraction, orthodontic treatments, pericoronitis and trauma were higher in 2020, *p* < 0.05 (treatment: 2019/2020; cementation: 1.3%/4.4%; oral surgery: 0.3%/1.5%; follow-ups: 4.8%/14.7%; endodontics: 6.3%/14.4%; oral medicine consultation: 0.03%/0.9%; tooth extraction: 4.9%/18.2%; orthodontic treatments: 0.8%/2.1%; pericoronitis: 0.2%/0.3%; trauma: 0.04%/0.3%) (Table 4).

## 4. Discussion

SARS-COV-2 spreads rapidly, causing COVID-19 epidemic outbreaks with a significant number of deaths worldwide [17], in addition to the potential collapse of healthcare systems. This displacement of other health priorities has been detrimental to other health problems, including non-communicable diseases [18]. The world quarantine was unprecedented, and it is inevitably expected to have a health effect. Equally in Argentina, having the whole country population under quarantine was unprecedented, and the subsequent impacts are unknown [3]. 

Most patients seen in emergency care clinics had moderate to severe pain associated with pulpal or periapical inflammation, oral infections, and trauma. Patients could not manage these types of pain with over-the-counter analgesics. Many patients required antibiotics or definitive treatment, such as tooth extraction, incision, drainage, or root canal therapy, to eliminate the disease and prevent the spread of infection. If dental emergency services had not been available, these patients would have likely visited the hospital emergency department for pain management, adding strains to the already overburdened emergency rooms due to COVID-19 and other medical emergencies. 

Our results showed a strong impact of the pandemic on the treatment demands of the population with significant modifications in the number of performed procedures. 

In both institutions, the number of patients seen in 2020 was several times lower than in 2019 during the same period of the year. It was reduced by about four times in the private clinic and five times in the university hospital. The number of treatments was also reduced in 2020 compared to 2019. In the clinic, it was reduced by almost four times; in the hospital, by nearly three times. Although the use of dental services during the pandemic was not intensely analyzed [7,8,19], several studies showed changes in patterns of health care service use for other diseases or emergency conditions. Hospital admissions for all major non-COVID-19 disease groups decreased during national lockdowns compared with the pre-pandemic baseline period [20,21], which could be explained by governmental policies that prioritized COVID-related healthcare services by restrictions, as well as other factors. A retrospective study comparing dental emergencies between pre-epidemic, epidemic, and post-epidemic periods found a drastic decrease in the number of patients visiting the stomatology department [22]. 

Papautsky et al. [23] reported that the most commonly reported barrier to receiving health care was the fear of SARS-CoV-2 infection, and among those who experienced health care delays one of the top three reported types of care that were affected was dental care. The risk of infection of health care workers in a dental setting should not be a barrier for emergency care if proper infection control measures are adopted. In a previous study performed at the University Dental Hospital of the University of Buenos Aires, we analyzed the prevalence of SARS-CoV-2 infection among dental professionals and auxiliaries and found lower values than the general population [24]. 

Caggeti et al. [8] described a reduction in the number of patients seeking urgent dental care inversely associated with epidemiologic conditions, both in females and males regardless of age. We found that, in both oral care centers, the distribution according to age group varied significantly between 2019 and 2020. However, the variation was more substantial in the private clinic, where the percentage of patients aged 60 years or more in 2020 was around a third of the percentage that represented the same age group in 2019. The relationship between year of care and age group was, although statistically significant, negligible within the university hospital. An internet-based, cross-sectional survey study [23] showed that age was significantly associated with experiencing health care delays, including dental treatments. Moffat et al. [25] found that older age was positively related to perceived susceptibility for contracting COVID-19 in a dental setting and reported that people felt most comfortable returning to dental offices following actions from public health experts, including vaccine development, easing of social distancing requirements, and appropriate recommendations.

Regarding the type of treatment delivered during the pandemic period, we found that the most frequent treatment performed was tooth extraction (23.4%) in the private clinic and radiographic examinations (30.9%) in the university hospital. Post hoc comparisons indicate that the percentages of all types of treatment differ significantly between the two sites (*p* < 0.05), except for oral surgery, oral medicine, pericoronitis, and traumatisms. The percentages of cementation, endodontic treatments, tooth extractions, orthodontics consultation, periodontic treatments, prosthetic treatments, and restorations were higher in the private clinic (*p* < 0.05). On the contrary, the percentages of general consultations, follow-ups, radiographic exams, and pediatric consultations were higher in the university hospital (*p* < 0.05). 

As in 2020, in 2019, a significant association was found between the provided treatment and the place of care, with a Cramer’s V coefficient of 0.78, which indicates a strong association. However, in this case, the most represented treatment in the private clinic was (25.6%), while in the hospital this was radiographic exams (59.9%). Post hoc comparisons indicate that the percentages of all treatments differ significantly between the two centers, except for oral surgery and traumatisms. The percentages of cementations, follow-ups, endodontic treatments, oral medicine consultations, tooth extraction, pediatric consultation, orthodontic treatments, pericoronitis, periodontic treatments, prosthetic treatments, and restorations were significantly higher in the private clinic. In contrast, the percentages of general consultations and radiographic exams were significantly higher in the university hospital. 

There are some limitations in our study that need to be considered. First, this study relied on a sample obtained only in two dental care institutions. However, most private clinics were closed, and dentists who worked in the public system were reassigned to develop other functions, which would have prevented including more dental care centers. Another limitation was constituted by the type of criteria used to group dental treatments. However, both care centers used the same kind of records allowing a better comparison of the results.

In our report, the number of emergency consultations performed both in the private clinic or the university hospital largely overpass previous studies and could help describe dental needs patterns in the pandemic period. 

In Argentina, a high percentage of the population engage in informal work with precarious income and social protection. Pandemic-associated economic problems could act as a barrier to fee-for-service health providers, including oral care. During the first stages of the pandemic, many health providers could not provide urgent care services to their patients due to staffing issues or lack of adequate PPEs. Fear of COVID-19 and other barriers should be explored to overcome the problems associated with differing treatments. Dental practitioners should aim to return the population to good oral health habits, while not increasing the spread of COVID-19. 

## 5. Conclusions

During the period of the year studied, the scenario generated by the pandemic led to a significant decrease in the usual number of patients and treatments. Although the two analyzed centers presented different characteristics concerning the distribution of treatments, the pandemic altered that distribution in both places. The pandemic also produced a change in the patients’ age groups, mainly in the private clinic. As the pandemic is still an ongoing sanitary problem in many countries, dental urgent care service is especially important. 

## Figures and Tables

**Table 1:** Distribution of patients according to age group.

	2019			2020		
Age (Years)	n	%	CI95 (%)	n	%	CI95 (%)
<9	769	2.1	2.0–2.3	283	3.8	3.4–4.2
10–19	2080	5.8	5.5–6.0	351	4.7	4.2–5.2
20–29	6837	19.0	18.6–19.4	1594	21.3	20.4–22.2
30–39	7172	19.9	19.5–20.3	1855	24.8	23.8–25.8
40–49	5996	16.6	16.2–17.0	1472	19.7	18.8–20.6
50–59	5290	14.7	14.3–15.0	998	13.3	12.6–14.1
60–69	3947	10.9	10.6–11.3	604	8.1	7.5–8.7
70–79	2718	7.5	7.3–7.8	261	3.5	3.1–3.9
>80	1262	3.5	3.3–3.7	67	0.9	0.7–1.1
Total	36,071	100		7485	100	

**Table 2:** Distribution of dental treatments performed in pandemic period (March–June 2020) according to dental care center.

Type of Treatment	Private Clinic		University Hospital	
	n	% (CI95)	n	% (CI95)
Emergency cementation of dental restorations *	328	**6.8 (6.1–7.5)**	298	3.2 (2.9–3.6)
Oral Surgery	60	1.2 (1.0–1.6)	150	1.6 (1.4–1.9)
General dental consultation *	92	1.9 (1.5–2.3)	1517	**16.3 (15.6–17.1)**
Follow-up *	514	10.6 (9.7–11.5)	1562	**16.8 (16.0–17.6)**
Endodontic treatments *	961	**19.8 (18.7–20.9)**	1073	11.5 (10.9–12.2)
Oral medicine consultation	30	0.6 (0.4–0.9)	93	1.0 (0.8–1.2)
Tooth extraction *	1138	**23.4 (22.3–24.6)**	1445	15.5 (14.8–16.3)
Radiography *	187	3.8 (3.3–4.4)	2874	**30.9 (30.0–31.8)**
Pediatric consultation *	14	0.3 (0.2–0.5)	93	**1.0 (0.8–1.2)**
Ortodoncia *	302	**6.2 (5.6–6.9)**	0	0.0 (0.0–0,0)
Pericoronitis	24	0.5 (0,.3–0.7)	24	0.3 (0.2–0,.4)
Periodontal treatments *	132	**2.7 (2.3–3.2)**	12	0.1 (0.1–0.2)
Prosthetic treatment *	379	**7.8 (7.1–8.6)**	56	0.6 (0.5–0.8)
Conservative treatment *	686	**14.1 (13.2–15.1)**	75	0.8 (0.6–1.0)
Traumatisms	11	0.2 (0.1–0.4)	31	0.3 (0.2–0.5)
Total	4858		9303	

Chi-square = 4514.53; *p* < 0.05; Cramer’s V = 0.56; (*) *p* < 0.05 *post-hoc.* Significant differences are highlighted in bold

**Table 3:** Distribution of dental treatments performed in pre-pandemic period (March–June 2019) according to dental care center.

Type of Treatment	Private Clinic		University Hospital	
	n	% (CI95)	n	% (CI95)
Emergency cementation of dental restorations *	348	**1.8 (1.6–2.0)**	245	1.0 (0.8–1.1)
Oral Surgery	60	0.3 (0.2–0.4)	88	0.3 (0.3–0.4)
General dental consultation *	2209	11.5 (11.1–12.0)	8382	**32.6 (32.1–33.2)**
Follow-up *	1735	**9.0 (8.6–9.4)**	408	1.6 (1.4–1.7)
Endodontic treatments *	2070	**10.8 (10.3–11.2)**	739	2.9 (2.7–3.1)
Oral medicine consultation	15	**0.1 (0.0–0.1)**	0	0.0 (0.0–0.0)
Tooth extraction *	1860	**9.7 (9.3–10.1)**	345	1.3 (1.2–1.5)
Radiography *	1266	6.6 (6.2–7.0)	15,383	**59.9 (59.3–60.5)**
Pediatric consultation *	443	**2.3 (2.1–2.5)**	4	0.02 (0.01–0.04)
Orthodontics consultation *	351	**1.8 (1.6–2.0)**	0	0.0 (0.0–0.0)
Pericoronitis	51	**0.3 (0.2–0.3)**	20	0.1 (0.1–0.1)
Periodontal treatments *	2410	**12.5 (12.1–13.0)**	3	0.01 (0.00–0.03)
Prosthetic treatment *	1461	**7.6 (7.2–8.0)**	50	0.2 (0.1–0.3)
Conservative treatment *	4920	**25.6 (25.0–26.2)**	12	0.05 (0.03–0.08)
Traumatisms	6	0.03 (0.01–0.07)	10	0.04 (0.02–0.07)
Total	19,205		25,689	

Chi-square = 27,128.36; *p* < 0,05; Cramer’s V = 0.78; (*) *p* < 0,05 *post-hoc.* Significant differences are highlighted in bold

**Table 4:** Distribution of patients according to year of treatment.

Type of Treatment	Year			
	2019 (Pre-Pandemic)		2020 (Pandemic)	
	n	% (CI95)	n	% (CI95)
Emergency cementation of dental restorations *	593	1.3 (1.2–1.4)	626	**4.4 (4.1–4.8)**
Oral Surgery	148	0.3 (0.3–0.4)	210	**1.5 (1.3–1.7)**
General dental consultation *	10,591	**23.6 (23.2–24.0)**	1609	11.4 (10.8–11.9)
Follow-up *	2143	4.8 (4.6–5.0)	2076	**14.7 (14.1–15.3)**
Endodontic treatments *	2809	6.3 (6.0–6.5)	2034	**14.4 (13.8–15.0)**
Oral medicine consultation	15	0.03 (0.02–0.06)	123	**0.9 (0.7–1.0)**
Tooth extraction *	2205	4.9 (4.7–5.1)	2583	**18.2 (17.6–18.9)**
Radiography *	16,649	**37.1 (36.6–37.5)**	3061	21.6 (20.9–22.3)
Pediatric consultation *	447	1.0 (0.9–1.1)	107	0.8 (0.6–0.9)
Orthodontics consultation *	351	0.8 (0.7–0.9)	302	**2.1 (1.9–2.4)**
Pericoronitis	71	0.2 (0.1–0.2)	48	**0.3 (0.3–0.4)**
Periodontal treatments *	2413	**5.4 (5.2–5.6)**	144	1.0 (0.9–1.2)
Prosthetic treatment *	1511	3.4 (3.2–3.5)	435	3.1 (2.8–3.4)
Conservative treatment *	4932	**11.0 (10.7–11.3)**	761	5.4 (5.0–5.8)
Traumatisms	16	0.04 (0.02–0.06)	42	**0.3 (0.2–0.4)**
Total	44,894		14,161	

Chi-square = 8407.20; *p* < 0.05; Cramer’s V = 0.38; (*) *p* < 0.05 *post-hoc.* Significant differences are highlighted in bold

## Data Availability

The data that support the findings of this study are available from the corresponding author upon reasonable request.
